# Bioavailability of Different Vitamin D Oral Supplements in Laboratory Animal Model

**DOI:** 10.3390/medicina55060265

**Published:** 2019-06-10

**Authors:** Egidijus Šimoliūnas, Ieva Rinkūnaitė, Živilė Bukelskienė, Virginija Bukelskienė

**Affiliations:** 1 Institute of Biochemistry, Life Sciences Center, Vilnius University, LT- 10257 Vilnius, Lithuania; egidijus.simoliunas@gmc.vu.lt (E.Š.); Ieva.rinkunaite@bchi.vu.lt (I.R.); 2 Public Institution Vilnius Centro Outpatient Clinic, LT-01117 Vilnius, Lithuania; zbukelskiene@gmail.com

**Keywords:** vitamin D, oral supplements, vehicle, bioavailability, vitamin D deficiency

## Abstract

*Background and Objectives:* The major cause of vitamin D deficiency is inadequate exposure to sunlight. It is difficult to supplement it with food because sufficient concentrations of vitamin D naturally occur only in a handful of food products. Thereby, deficiency of this vitamin is commonly corrected with oral supplements. Different supplement delivery systems for improved vitamin D stability and bioavailability are proposed. In this study, we compared efficiency of three vitamin D delivery systems: microencapsulated, micellized, and oil-based. *Materials and Methods:* As a model in this medical testing, laboratory rats were used for the evaluation of bioavailability of different vitamin D vehicles. Animals were divided into three groups: the first one was given microencapsulated vitamin D_3_, the second—oil-based vitamin D_3_, and the third—micellized vitamin D_3_. Test substances were given *per os* to each animal for 7 days, and vitamin D concentration in a form of 25-hydroxyvitamin D (25(OH)D) in the blood was checked both during the vitamin delivery period and later, up to the 24th day. *Results:* Comparison of all three tested products showed that the microencapsulated and oil-based vitamin D_3_ vehicles were the most bioavailable in comparison to micellized vitamin D_3_. Even more, the effect of the microencapsulated form of vitamin D_3_ remained constant for the longest period (up to 14 days). *Conclusions:* The results of this study suggest that the oral vitamin D supplement vehicle has an impact on its bioavailability, thus it is important to take into account how much of the suppled vitamin D will be absorbed. To maximize the full exploit of supplement, the best delivery strategy should be employed. In our study, the microencapsulated form of vitamin D was the most bioavailable.

## 1. Introduction

**Figure 1:**
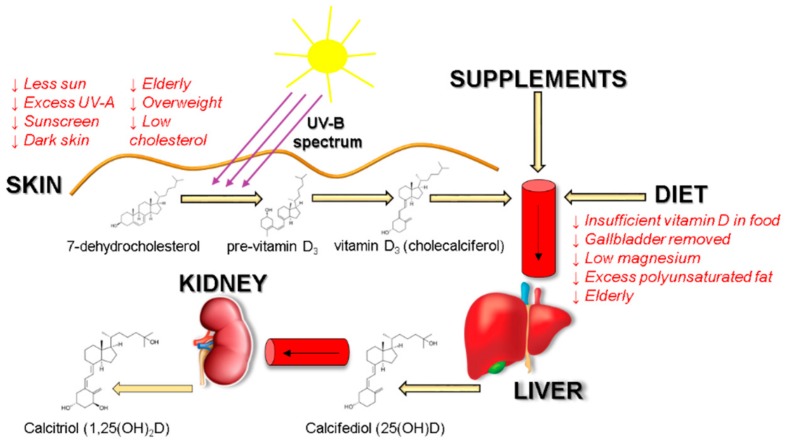
Vitamin D_3_ sources, biosynthesis and possible factors affecting absorption. Downward arrows show factors that are associated with decreased vitamin D absorption and synthesis in the organism.

Vitamin D deficiency is a global health issue that afflicts more than 1 billion children and adults worldwide. The vitamin D deficiency has been associated with many acute and chronic illnesses, including preeclampsia, childhood dental caries, periodontitis, autoimmune disorders, infectious diseases, cardiovascular diseases, various types of cancer, type 2 diabetes, and neurological disorders [1]. Although vitamin D can be synthesized upon exposure to sun, inadequate amount of sunlight is one of the major causes for the pandemic of vitamin D deficiency [2,3]. During sun exposure, 7-dehydrocholesterol, the immediate precursor in the cholesterol biosynthetic pathway, absorbs ultraviolet B radiation (290–315 nm) resulting in breaking of the bond between carbon 9-carbon 10 atoms to produce pre-vitamin D_3_. Once formed, this thermodynamically unstable second steroid undergoes a rearrangement of its triene system to form the thermodynamically stable vitamin D_3_, which is then transported to the liver and converted to 25-hydroxyvitamin D 25(OH)D. This metabolite then re-enters the circulation and travels to the kidneys, where it is converted to the active form 1,25-dihydroxyvitamin D, also known as calcitriol—the hormonally active metabolite [4,5] (Figure 1).

Vitamin D can also be obtained from diet, however, very few food products naturally contain a sufficient amount of vitamin D. Among the richest are the following products: oily fish, in particular salmon, mackerel, and herring; some mushrooms; cod liver oil; eggs; and dairy products [6,7]. Some and sometimes substantial amounts of vitamin D (25(OH)D) are present in meat, including beef and pork [8,9]. By adding 25(OH)D into animal feed, an increased amount of vitamin D_3_ in poultry, pork, and beef can be achieved [10].

Multiple factors might affect vitamin D levels: age, being indoors, dark skin, sunscreen use, and low cholesterol might impede with vitamin D_3_ biosynthesis in skin. Dietary vitamin D_3_ bioavailability is greatly reduced upon gallbladder removal; gastrointestinal diseases, such as Crohn’s; cystic fibrosis; and celiac disease. Low magnesium intake might lead to an insufficient amount of active vitamin D_3_ as magnesium assists in the activation of vitamin D [11]. Correction of vitamin D insufficiency is commonly achieved using oral vitamin D supplements. The Endocrine Society guidelines suggest that daily intake of 1500 to 2000 international units (IU) of vitamin D is necessary to achieve serum 25(OH)D concentrations consistently >30 ng/mL in adults to prevent vitamin D deficiency [12,13]. Studies using radiolabeled vitamin D_3_ showed that its absorption efficiency varies between 55% to 99%, however, it does not depend upon fat content consumed with food, yet lipid composition impacts vitamin D bioavailability [14,15,16]. Long chain fatty acids interfere with vitamin D absorption [17]. Absorption efficiency of vitamin D is also increased by supplements in which vitamin D is placed into micelles, microcapsules, or liposomes. Emulsification of drugs or nutrients and their insertion into micelles or microcapsules have many benefits: higher stability to aggregation and gravitational separation; higher optical clarity; protection from degradation, light, and oxidation; and improved bioavailability of water insoluble and difficultly absorbed compounds [18,19]. These delivery vehicles protect vitamin D from the environment [18]. Encapsulation mimics naturally occurring process—during digestion, vitamin D is transferred from its food matrix to the mixed micelles generated by the lipolysis of dietary fat carried out by bile salts [20]. Enterocytes use SR-BI (scavenger receptor class B type I), CD36, and NPC1L1 (Niemann-Pick C1-Like 1) membrane transporters to endocytose these particles. Thus, by packing vitamin D in lipid vesicles, active vitamin D absorption can be exploited and facilitated. In this research, we tested vitamin D bioavailability dependence to oral supplement delivery system used—micellar and microencapsulated drug delivery systems were compared to the standard oil-based preparation.

## 2. Materials and Methods

### 2.1. Test Substances

SmartHit IV™ microencapsulated vitamin D_3_ is an oral supplement containing 2000 IU/mL cholecalciferol and natural lecithin composing microcapsule. It is a water-soluble form of vitamin D where an active substance is loaded in microcapsules.

Micellized vitamin D_3_ is an oral supplement containing 15,000 IU/mL cholecalciferol. It contains macrogolglycerol ricinoleate—a synthetic, nonionic surfactant that stabilizes emulsions of nonpolar materials in water. It is a water-soluble form of vitamin D that is encapsulated in nanodispersed micelles.

Oil-based vitamin D_3_ is an oral supplement containing 400 IU/drop cholecalciferol. According to manufacturers, one drop contains 35 uL of volume. It is an oil-based form of vitamin D. 

All the test substances were purchased in the local pharmacy. Their descriptions were obtained from the pharmaceutical instructions.

### 2.2. Animals

Wistar rats (7–9 weeks old; Vilnius University; Lithuania; License of Animal Ethics Committee No G2-47, 30 June 2016) weighing 160–200 g were maintained under standard conditions: Temperature +22 ± 1 °C, humidity 55 ± 3%, and 12 h/12 h light-dark cycle. They were fed and watered ad libitum with standard commercial rodent feed JE-83004920 (Joniškio grūdai, Ltd., Joniškis, Lithuania), watered with tap water.

### 2.3. Experimental Design

**Figure 2:**
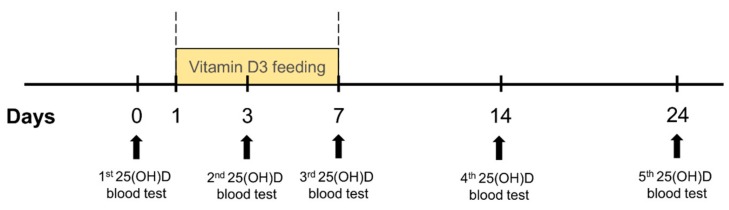
General experimental design. In experiment, rats were randomly divided into three groups (*n* = 6) by supplement given. After baseline 25-hydroxyvitamin D 25(OH)D blood test, rats were given vitamin D_3_ supplements for 7 consecutive days. Blood was then drawn at the 3rd, 7th, 14th, and 24th days of the experiment.

The animal experiment was designed in accordance to the requirements stated in 2010/63/EU Directive and Order of the Lithuanian State Food and Veterinary Service Director No B1-866; 31 December 2012. All procedures were approved by License of Animal Research Ethics Committee (Lithuania) No G2-47, 2016-06-30. Rats were randomly divided into 3 groups (6 animals per group) and singly housed in a cage. Following 3 days after grouping, before giving test substances, from each rat’s tail vein, 500 μL of blood was taken and blood serum was separated. In this way, the control level (“0 day”) of each animal’s 25(OH)vitamin D concentration was determined. The first group was given microencapsulated, the second—oil-based, and the third—micellized vitamin D_3_. Test substances were given *per os* to each animal for 7 days each morning. Blood samples were collected before the daily dosing at the 3rd and 7th day starting from the beginning of the experiment (during the vitamin D_3_ feeding period). From the 8th day, the delivery of the test products was terminated, but the vitamin D_3_ concentration in the blood was checked again at the 14th and 24th day (from the start of the experiment) (Figure 2).

### 2.4. Preparation of Test Substances

The test substances were prepared in such a way that each rat per day received 2000 IU/kg vitamin D_3_. Oil-based supplement was diluted in olive oil, micellized vitamin D_3_ was diluted in tap water, and SmartHit IV™ microencapsulated form was not diluted and was given as originally presented.

### 2.5. Preparation and Analysis of Blood Serum

After collection of whole blood, the samples were left undisturbed at room temperature for 3 h. Then, the emerging clot was separated from the wall of the tube, and the samples were placed into the refrigerator (+4 °C). After 18 h, the clot was removed by centrifuging for 15 min at 1500 rpm in a refrigerated centrifuge. The resulting supernatant was the serum (150–200 μL). It was collected into clean tubes and immediately used for the determination of vitamin D_3_ concentration. The amount of 25(OH)D in blood serum was analyzed by electrochemiluminescence immunoassay (ECLIA) using Cobas^®^ 6000 modular analyzer (Roche, Indianapolis, USA).

### 2.6. Statistical Analysis

Data analysis was performed using the RStudio statistical analysis program (version 1.1.453). Differences between the levels of vitamin D in animal blood serum on the respective day of the study were evaluated by a single factor ANOVA. The vitamin D bioavailability was assessed by calculation of area under the curve (AUC) method. Obtained significant differences were identified using Tukey LSD *post hoc* test. Data were considered statistically significantly different if *p* < 0.05. The data are shown by boxplot and dotted graphs. Statistically significant differences in the graphs are marked with asterisks (*); *—when *p* < 0.05, **—when *p* < 0.01.

## 3. Results

**Figure 3:**
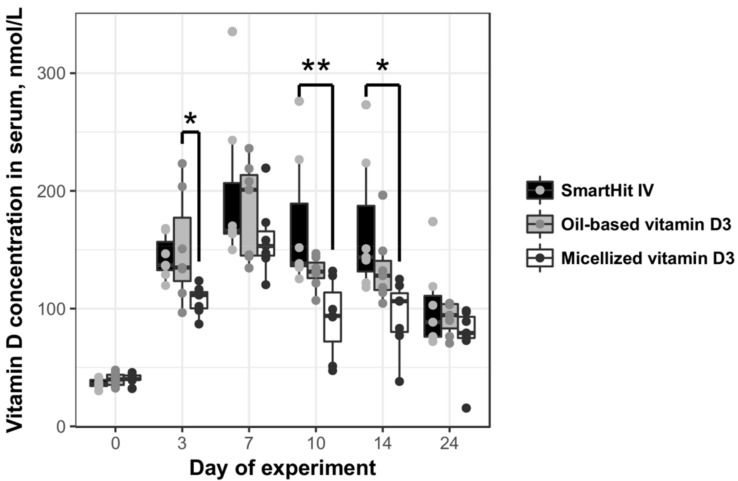
Vitamin D_3_ concentration in rat blood serum during 24 days of experiment: rats were divided into three groups; from the 1st to 7th day, each rat in the group was orally given 2000 international units (IU)/kg vitamin D_3_ presented in different forms: microencapsulated SmartHit IV™, oil-based, and micellized.

According to our data, amounts of vitamin D_3_ increased in the blood serum of all treated animal groups in proportion to time, during vitamin supplementation, until the 7th day (Figure 3). As early as after 3 days of supplementation, microencapsulated and oil-based vitamin D_3_ increased vitamin levels in the blood by almost three times: The control level of vitamin D_3_ in the rat serum ranged from 36.49 ± 4.12 to 40.5 ± 3.05 nmol/L, meanwhile in the microencapsulated and oil-based treatment groups it got up to 143.35 ± 14.72 and 150.85 ± 35.77 nmol/L, respectively. The highest vitamin D_3_ concentration in the rat blood serum was registered in the oil-based vitamin D_3_ group on day 7—the tested vitamin concentration reached 198.93 ± 51.6 nmol/L. Comparing the duration of the effect of all vitamin vehicles, microencapsulated SmartHit IV™ supplementation vitamin D_3_ concentrations in the blood serum remained constant for the longest time (up to the 14th day).

AUC analysis confirmed that the least bioavailable delivery system was micellized vitamin D_3_. It was absorbed almost twice as inefficiently in comparison to microencapsulated vitamin D_3_ (Table 1). Additionally, the fat-soluble form of vitamin D_3_ was also more bioavailable to rats than micellized vitamin D_3_. Vitamin D_3_ packed in microcapsules was the most bioavailable.

## 4. Discussion

Vitamin D has the unique property of being synthesized in the skin from the exposure to sunlight. However, in Northern regions, due to the lack of sunlight, vitamin D deficiency is widespread among children and adults [2,5,7,10]. This is further worsened by the insufficient vitamin D-rich food consumption [9,10]. Thus, correction of vitamin D deficiency is commonly achieved using oral vitamin D supplements [12]. Vitamin D is a fat-soluble molecule, which when ingested, dissolves in dietary fat, is emulsified by the bile salts, and is absorbed by intestinal enterocytes. The absorption efficiency or bioavailability depends on lipid composition and food supplement vehicle. Therefore, there has been an increased interest in optimizing supplementation strategies, especially in populations at risk of vitamin D insufficiency, such as the elderly; obese individuals; and people with certain chronic diseases, such as Crohn’s or chronic kidney disease [21,22]. Usually, water-insoluble nutrients are dissolved in lipid-based solvents. Commercially available solubilized oral formulations include various natural oils: peanut, corn, soybean, sesame, olive [23]. A systematic review on vitamin D supplement bioavailability showed that oil-soluble vehicles produce greater increase of 25(OH)D in blood serum when compared to powder- and ethanol-based supplements [24].

Some substances, such as microemulsions, microcapsules, liposomes, and micelle-forming compounds are used to further improve absorption efficiency of fat-soluble vitamins. Micellization is a delivery system of fat-soluble nutrients that are micronized into water-soluble micellar spheres using surfactants, such as Polysorbate 80 (also known as Tween 80), citric acid monoglyceride esters, and polyethylene glycol 400 (PEG 400). Surfactants self-assemble and form micelles once the surfactant monomer concentration reaches the critical micelle concentration—a concentration of a surfactant in a bulk phase, above which micelles start to form. Vitamin D_3_ used in this study was micellized using macrogolglycerol ricinoleate, also known as Cremophor EL, which is a synthetic, non-ionic surfactant used to emulsify and solubilize water-insoluble materials in water [23,25]. Supplement manufacturers recommend using their supplements with food or beverages. Micellized product contains macrogolglycerol ricinoleate, which ensures micelle stability in water, according to the supplement leaflet. Micelles must be stable enough to not release a cargo upon oral or systemic administration and must remain intact long enough to transport nutrients or drugs to the target site.

SmartHit IV™ vitamin D_3_ microcapsules are composed of natural phospholipid bilayer that encapsulates a fraction of the surrounding aqueous medium. Due to biphasic nature, microcapsules can contain both lipid-soluble and water-soluble components. Microcapsules protect the nutrients from degradation in acidic pH found in the stomach (pH 1.4–4.0), and in turn encapsulated substances tend to stay inside the microcapsules and thus do not cause stomach irritation [26]. Nutrients that are sensitive to oxygen and sunlight are protected due to microencapsulation technology. Most importantly, microcapsules improve the absorption and bioavailability of nutrients up to five times in comparison to their free counterparts [27]. In this study, we compared three different vehicles—microcapsules, micelles, and lipids. Rats were able to absorb 25% more vitamin D that was microencapsulated in comparison to the most commonly used oil-based supplementation. Although some studies show micellar nutrients to be absorbed better than their oil-based, liposomal, or ethanol dissolved counterparts [24,28], in our study, rats were able to absorb only about 65% of vitamin D in comparison to the oil-based vehicle, and it also was almost two times less bioavailable compared to SmartHit IV™ microcapsules.

This pre-clinical *in vivo* research laid basis for clinical study on human volunteers with vitamin D deficiency, which would be conducted in the near future. Currently, using animal models is the best choice for quick comparison of the efficiency of different vitamin D vehicles. It is known that rats and humans share a majority of their biochemical capabilities at the genome level, which gives an important role for rats by considering them as a model organism for understanding human biology and diseases. In terms of the expressions of vitamin D-metabolizing enzymes and vitamin D receptors in rats, it should be noted that they are similar to those in humans [29]. However, despite an extremely small number of species-specific differences at the genome level, individual differences at the gene-level can alter some functions. Therefore, the results of this research provided the basis for further studies involving human volunteers with vitamin D deficiency.

## 5. Conclusions

Comparison of three tested products showed that the microencapsulated and oil-based vitamin D_3_ supplement delivery systems could be characterized by their better bioavailability in comparison to micellized vitamin D_3_. Further, the effect of microcapsulated vitamin D supplement remained constant for the longest time, even for seven days after supplementation, showing its prolonged effect. Although all the tested products have increased vitamin D concentration in rats’ blood serum significantly and could be used to treat or prevent vitamin D deficiency, the most efficient was SmartHit IV™ microcapsulated vitamin D_3_ supplement.

## Figures and Tables

**Table 1:** Area under the curve pharmacokinetics

Group	AUC	AUC_x_/AUC_Oil-Based_	C_Max_ Vitamin D Concentration in Blood Serum, nmol/L	Day of Maximum Concentration of Vitamin D
**SmartHit IV™**	2651.00	1.24	335.4	7
**Oil-based vitamin D_3_**	2135.60	1.00	236.1	7
**Micellized vitamin D_3_**	1378.45	0.65	216.4	7
